# Genome-Wide Identification and Expression Profiling of the GRAS Gene Family in *Taxus cuspidate*

**DOI:** 10.3390/genes16111345

**Published:** 2025-11-07

**Authors:** Li Gao, Aokun Shi, Mian Wang, Hui Tian, Yanwen Zhang

**Affiliations:** 1 Department of Seed Science and Engineering, Liaodong University, Dandong 118003, China; gaoli@liaodongu.edu.cn (L.G.); tianhui@liaodongu.edu.cn (H.T.); 2 Beijing Tianhaoyuan Agricultural Technology Development Co., Ltd., Beijing 100081, China

**Keywords:** *Taxus cuspidata*, GRAS gene family, genome-wide identification, phylogenetic analysis, expression pattern analysis

## Abstract

Background: The GRAS transcription factor family plays pivotal regulatory roles in plant growth and development, hormone signaling, and responses to environmental stresses. *Taxus cuspidata*, an endangered and nationally protected conifer species endemic to China, is recognized as the sole natural source of paclitaxel—an anti-cancer compound of considerable pharmaceutical importance. While GRAS family genes have been systematically characterized in various plant species, a comprehensive investigation in *T. cuspidata* has yet to be conducted. Methods: In this study, 26 *TcGRAS* genes were identified and characterized through genome-wide analysis. Results: Phylogenetic analysis grouped these genes into seven subfamilies, indicating a high degree of evolutionary conservation. Gene structure analysis revealed that most *TcGRAS* genes lacked introns, while promoter regions were enriched with cis-acting elements related to light responsiveness, hormone signaling (e.g., MeJA, ABA, SA), and abiotic stress responses. Tissue-specific expression profiling and qRT-PCR analyses showed that several *TcGRAS* genes were highly expressed in paclitaxel-accumulating organs such as roots, stems, and bark—particularly TcGRAS6, TcGRAS13, and TcGRAS24—suggesting their putative involvement in paclitaxel biosynthesis through hormone-mediated regulatory pathways. Co-expression network analysis further identified TcGRAS13 and TcGRAS14 as central nodes within the transcriptional regulatory network. Conclusions: This study provides the first genome-wide identification and comprehensive characterization of the GRAS gene family in *T. cuspidata*, thereby establishing a theoretical framework and providing valuable candidate gene resources for elucidating their biological functions and regulatory roles in secondary metabolism, particularly in paclitaxel biosynthesis.

## 1. Introduction

Throughout their life cycle, plants are constantly exposed to and must adapt to a wide range of dynamic environmental fluctuations, including changes in light, water availability, temperature, and biotic stressors. To efficiently perceive and respond to both internal and external cues, plants employ a complex and tightly regulated transcriptional network to coordinate growth, developmental processes, and environmental adaptation [1]. Within this regulatory framework, transcription factors function as central regulators by specifically recognizing and binding to cis-acting elements in the promoters of target genes, thereby facilitating the precise spatiotemporal control of downstream gene expression [2].

The GRAS gene family comprises plant-specific transcription factors, named after its first three identified members: GAI (GA-Insensitive), RGA (Repressor of ga1-3), and SCR (Scarecrow) [3,4]. GRAS proteins are defined by a highly conserved C-terminal GRAS domain, which contains several signature motifs—LHRI, VHIID, LHRII, PFYRE, and SAW—that mediate protein–protein interactions and transcriptional activation or repression [5]. In contrast, the *N*-terminal region exhibits considerable structural variability, which is implicated in signal perception, protein complex assembly, and the specific recognition of target genes [6], thereby contributing to their functional diversification.

Extensive studies have shown that the GRAS gene family participates in a wide array of fundamental physiological processes across diverse plant species, including gibberellin (GA) signaling [7], root and stem differentiation, light signaling responses [8], hormonal crosstalk, and responses to both biotic and abiotic stresses. These findings underscore the extensive functional diversity and high degree of evolutionary conservation of this gene family. Among its subgroups, members of the DELLA subfamily, such as GAI and RGA, function as negative regulators in the GA signaling pathway, thereby suppressing excessive stem elongation and influencing plant height and branching architecture [9]. In contrast, members such as SCR and SHR are involved in regulating radial patterning in vascular and root tissues and serve as integral components of the root developmental network [10,11]. Within the GRAS family, the PAT1 subfamily has attracted significant interest owing to its critical involvement in light signal transduction. PAT1 proteins were first identified in *Arabidopsis thaliana* as positive nuclear regulators of the phytochrome A (phyA) signaling pathway, which activate downstream light-responsive genes and ultimately regulate photomorphogenesis [12,13]. Recent studies have further revealed that members of the PAT1 subfamily play pivotal roles in abiotic stress responses, such as those triggered by drought [14] and low temperatures [15], emphasizing their essential contribution to stress-responsive signaling networks. Moreover, emerging evidence indicates that PAT1 genes are functionally significant in plant grafting. Notably, their expression is significantly upregulated at the graft junctions of both *Arabidopsis* and *Picea abies*, indicating a pivotal role in wound healing and tissue regeneration during graft union formation [16].

Northeast yew (*Taxus cuspidata* Sieb. et Zucc.), a rare gymnosperm belonging to the family Taxaceae, is recognized as a valuable medicinal resource due to its production of paclitaxel, a compound extensively utilized in anti-cancer drug development and clinical therapy [17]. The biosynthesis of paclitaxel involves the coordinated expression of multiple genes and the catalytic activities of pathway-specific enzymes. To date, approximately 20 enzymatic steps have been proposed; however, the complete biosynthetic pathway remains to be fully elucidated [18]. The natural abundance of paclitaxel in *Taxus* species is extremely low, and the intrinsically slow growth rate of *T. cuspidata* further limits the sustainable production and supply of this compound. The recent completion of whole-genome sequencing for *T. cuspidata* has yielded valuable genomic resources that facilitate gene cloning, functional characterization, and comprehensive bioinformatic analyses [19].

Although the GRAS gene family has been characterized in numerous plant species, a comprehensive genome-wide characterization of this family in *T. cuspidata* species has yet to be reported. Whether *GRAS* genes contribute to paclitaxel biosynthesis or are involved in the unique tissue development and environmental adaptation characteristic of *T. cuspidata* remains unknown. In this study, members of the GRAS gene family in *T. cuspidata* were systematically identified on a genome-wide scale. Phylogenetic relationships, conserved domains, cis-regulatory elements, and tissue-specific expression patterns were comprehensively analyzed to infer their potential biological roles. These findings are expected to provide a foundational framework for future functional investigations and genetic improvement, as well as candidate gene resources for regulatory research and genetic engineering of paclitaxel biosynthesis.

## 2. Materials and Methods

### 2.1. Identification of TcGRAS Gene Family Members

The genome and annotation files of *T. cuspidata* were downloaded from the Ensembl Plants database (https://plants.ensembl.org/index.html) (accessed on 11 June 2025), and both protein and coding DNA (CDS) sequences were extracted using TBtools-II. The hidden Markov model (HMM) profile for the GRAS domain (PF03514) was obtained from the Pfam database. Candidate TcGRAS proteins were screened using the Pfam (https://pfam.xfam.org/), SMART (http://smart.embl-heidelberg.de/) (accessed on 14 June 2025), and CDD (https://www.ncbi.nlm.nih.gov/Structure/bwrpsb/bwrpsb.cgi) (accessed on 16 June 2025) databases to verify the presence of conserved GRAS domains. Genes lacking the characteristic GRAS domain were excluded from further analysis. The physicochemical properties of the identified TcGRAS proteins, including amino acid length, molecular weight, theoretical isoelectric point (pI), and hydrophobicity, were analyzed using the Protein Parameters module in TBtools-II.

### 2.2. Chromosomal Localization of TcGRAS Genes

Chromosomal positions and corresponding gene density data for TcGRAS family members were obtained from the GFF3 annotation file of *T. cuspidata* using TBtools-II. The chromosomal distribution of *TcGRAS* genes was visualized using TBtools-II. In addition, all TcGRAS genes were uniformly renamed according to their chromosomal positions.

### 2.3. Gene Structure and Conserved Motif Analysis of TcGRAS Genes

The gene structures of *TcGRAS* genes were analyzed and visualized using the Gene Structure Display module in TBtools-II, based on the GFF3 annotation file of *T. cuspidata*. Conserved motifs within the TcGRAS proteins were identified using the MEME online suite (http://meme.nbcr.net/meme/intro.html) (accessed on 25 June 2025), with motif widths set between 6 and 15 amino acids and the number of motifs limited to 55.

### 2.4. Phylogenetic Analysis of TcGRAS Genes

A rooted neighbor-joining (NJ) phylogenetic tree was constructed using MEGA 7.0 to analyze the evolutionary relationships and classification of GRAS proteins from *T. cuspidata*, *A. thaliana*, *Solanum lycopersicum* (tomato), and *Populus trichocarpa*. Protein sequences for *A. thaliana*, *S. lycopersicum*, and *P. trichocarpa* were retrieved from the UniProt database (https://www.uniprot.org/) (accessed on 2 July 2025). The resulting phylogenetic tree was exported in Newick format and visualized via the Chiplot online platform (https://www.chiplot.online/#).

### 2.5. Cis-Acting Element Analysis of TcGRAS Genes

The 2000 bp sequences upstream of the start codon (ATG) of all *TcGRAS* genes were extracted using TBtools-II. Cis-acting regulatory elements in the promoter regions were predicted via the PlantCARE database (http://bioinformatics.psb.ugent.be/webtools/plantcare/html/) (accessed on 4 July 2025), and the results were visualized using the ggplot2 v.4.0 package in R [20].

### 2.6. Expression Pattern and Functional Annotation Analysis of TcGRAS Genes

Transcriptomic data from five different tissue types—bark, leaves, roots, stems, and twig bark—were obtained from the NCBI database under a publicly available transcriptome project (BioProject: PRJNA661543). Three independent biological replicates were included for each tissue type to ensure data reliability and statistical power. All transcriptomic datasets were subjected to normalization and visualized using TBtools-II. To investigate the potential biological functions of the *TcGRAS* gene family, GO annotation was conducted using the GOATOOLS toolkit (https://github.com/tanghaibao/GOatools) (accessed on 24 July 2025). GO term information was obtained from the Gene Ontology Consortium database. During GO enrichment analysis, statistical significance for each GO term was evaluated via Fisher’s exact test, followed by multiple testing correction using the Bonferroni method. GO terms with *p* < 0.05 were considered significantly enriched and indicative of key biological processes associated with *TcGRAS* genes [21].

### 2.7. qRT-PCR Analysis

Nine healthy two-year-old *T. cuspidata* plants were randomly selected from the Jingou Science and Technology Ecological Park in Dandong, Liaoning Province, China, for quantitative real-time PCR (qRT-PCR) analysis. Bark, leaves, roots, stems, and young branch bark tissues were collected separately. For each tissue type, samples from every three plants were combined to form one biological replicate, resulting in a total of three biological replicates (*n* = 3). All samples were immediately flash-frozen in liquid nitrogen after collection and stored at –80 °C for subsequent analyses.

Total RNA was extracted using the RNAprep Pure Plant Kit (Tiangen, Beijing, China) according to the manufacturer’s instructions. First-strand cDNA was synthesized using the HiScript^®^ II 1st Strand cDNA Synthesis Kit (Vazyme, Nanjing, China). Quantitative real-time PCR was conducted using the Gentier 96E real-time PCR system (Tianlong Technology Co., Ltd., Xi’an, China) with the SYBR Green Premix Pro Taq HS qPCR Kit III (Accurate Biotechnology (Hunan) Co., Ltd., Changsha, China). Each cDNA sample was analyzed in three technical replicates. Relative gene expression levels were calculated according to the 2^−ΔΔCt^ method. All primer sequences are provided in Supplementary Table S1 and were synthesized by Bioengineering Co., Ltd. (Shanghai, China).

### 2.8. Data Analysis

Data collation and computation were performed using Microsoft Excel 2022. One-way analysis of variance (ANOVA) was conducted in SPSS 22.0 to evaluate statistical significance in gene expression across different groups at *p* values of <0.05, <0.01, <0.001, and <0.0001. Graphs were constructed and visualized using GraphPad Prism 8, R, and Adobe Illustrator 2023.

## 3. Results

### 3.1. Genome-Wide Identification and Physicochemical Analysis of GRAS Genes in T. cuspidata

Based on a combination of domain prediction and BLAST v.2.17 alignment against the NCBI database, a total of 26 GRAS gene members were identified in *T. cuspidata* after manually removing redundant sequences. To systematically characterize the structural and physicochemical properties of this gene family, a set of basic parameters was calculated for the 26 predicted TcGRAS proteins, including amino acid length, molecular weight (MW), theoretical isoelectric point (pI), instability index, hydropathicity (GRAVY), and aliphatic index (Table 1). The TcGRAS proteins ranged in length from 132 to 853 amino acids and had molecular weights ranging from 14.3 kDa to 92.1 kDa. The theoretical pI values ranged from 4.23 to 7.17, with most proteins showing pI values below 6.0. Additionally, 14 proteins were predicted to have instability indices exceeding 40. Analysis of hydropathicity revealed that most TcGRAS proteins were hydrophilic.

### 3.2. Chromosomal Localization of TcGRAS Gene Genes

**Figure 1:**
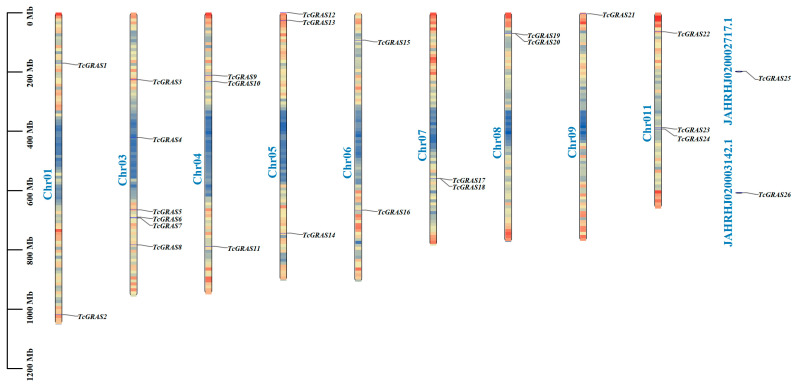
Chromosomal localization of TcGRAS family members in *T*. *cuspidate*. The vertical axis indicates chromosomal length. “chr01” to “chr011” represent the 11 chromosomes of *T. cuspidata*, with black labels indicating the positions of the 26 *GRAS* genes on the respective chromosomes.

Chromosomal mapping was performed to determine the genomic positions and physical distributions of the 26 identified *TcGRAS* genes in *T. cuspidata* (Figure 1). The results revealed that *TcGRAS* genes were unevenly distributed across 9 of the 11 chromosomes. Chromosome 3 contained the highest number of genes, with six members (TcGRAS3–TcGRAS8) clustered together. Chromosomes 4, 8, and 11 each harbored three *TcGRAS* genes, while chromosomes 1, 5, 6, 7, and 9 harbored one or two genes. Additionally, three genes (*TcGRAS25*, *TcGRAS26*, and *TcGRAS23*) were assigned to unplaced scaffold sequences (e.g., JAHRHJ020002717.1 and JAHRHJ020003142.1), suggesting that their genomic locations remain unanchored to specific chromosomes.

### 3.3. Phylogenetic Analysis of TcGRAS Genes

**Figure 2:**
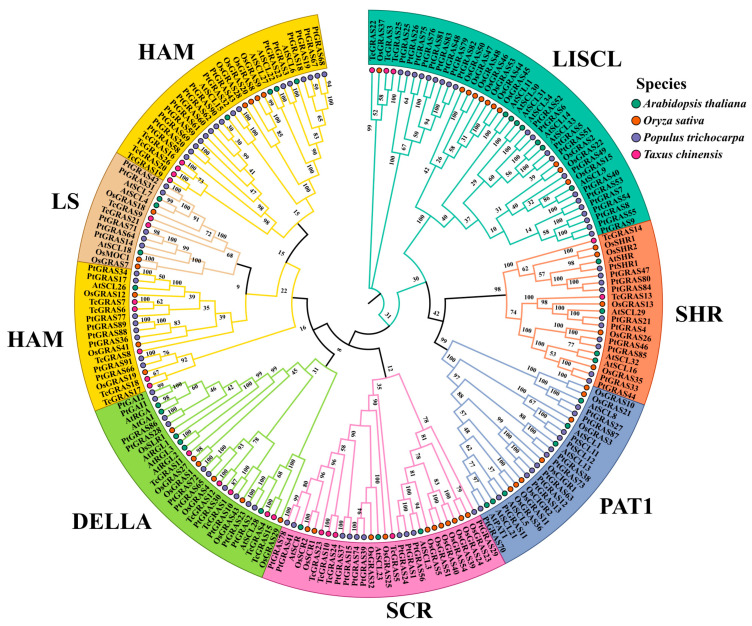
Phylogenetic relationships of GRAS family genes in *A. thaliana*, *O. sativa*, *P. trichocarpa*, and *T*. *cuspidate*. Four symbols and colors on the right under species denote the GRAS family members corresponding to *A. thaliana*, *O. sativa*, *P. trichocarpa*, and *T*. *cuspidata*. Branches corresponding to each subfamily are depicted in distinct colors, with those of members within the same subfamily shown in identical colors.

A phylogenetic tree was constructed to systematically examine the evolutionary relationships and functional diversification of the GRAS transcription factor family across diverse plant species, including *A. thaliana*, *Oryza sativa*, *P. trichocarpa*, and *T. cuspidata* (Figure 2). According to the classification system established for *A. thaliana*, the 26 *TcGRAS* genes were assigned to six well-defined subfamilies: LISCL, SHR, SCR, DELLA, HAM, and LS. Each subfamily formed well-supported monophyletic clades, indicating a high degree of phylogenetic conservation. Specifically, the LISCL subfamily included TcGRAS3, TcGRAS22, and TcGRAS25; the SHR subfamily included TcGRAS13 and TcGRAS14; the SCR subfamily included TcGRAS5, TcGRAS10, TcGRAS23, and TcGRAS24; and the HAM subfamily included TcGRAS6, TcGRAS7, TcGRAS8, TcGRAS16, TcGRAS17, TcGRAS18, TcGRAS19, TcGRAS20, and TcGRAS26. The DELLA subfamily comprised TcGRAS1, TcGRAS4, TcGRAS11, TcGRAS12, and TcGRAS15. Finally, the LS subfamily included TcGRAS9 and TcGRAS21. These findings suggest that several functionally defined core subfamilies have been evolutionarily conserved in *T. cuspidata*.

### 3.4. Motif, Conserved Domain, and Gene Structure Analysis of TcGRAS Genes

**Figure 3:**
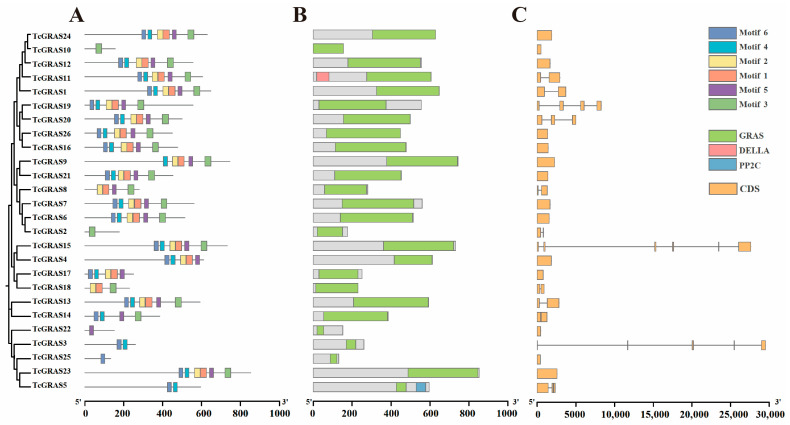
Motif, conserved domain, and gene structure analysis of TcGRAS family members. (**A**) Conserved protein motifs. (**B**) Structural domains. (**C**) Gene structures. The horizontal axis indicates the gene and protein sequence lengths.

A comprehensive analysis was performed to provide insights into the structural characteristics and conserved functions of the GRAS gene family in *T. cuspidata*. Six conserved motifs (Motif 1–6) were identified using the MEME suite (Figure 3A). Most TcGRAS proteins contained Motifs 1, 2, 3, and 5, whereas Motif 6 was uniquely detected in a limited subset of proteins, including TcGRAS10, TcGRAS11, and TcGRAS15. Domain architecture analysis revealed that all TcGRAS proteins possessed a canonical GRAS domain (Figure 3B), while several also contained additional domains such as DELLA and PP2C. Gene structure analysis showed that most *TcGRAS* genes contained either a single intron or were intronless (Figure 3C), as exemplified by TcGRAS1, TcGRAS9, and TcGRAS14. In contrast, a limited number of genes—such as TcGRAS5, TcGRAS23, and TcGRAS25—contained multiple introns.

### 3.5. Cis-Acting Elements Analysis of TcGRAS Genes

**Figure 4:**
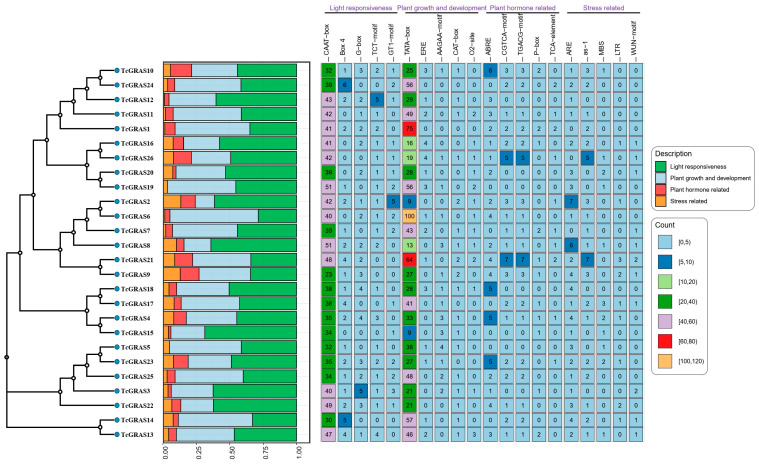
Distribution of cis-regulatory elements in the promoter regions of *TcGRAS* genes. The left vertical axis lists the 26 *TcGRAS* gene identifiers. The central stacked bar chart displays the total number of cis-acting elements classified by functional categories. The heatmap on the right shows representative cis-acting elements along the horizontal axis, with the frequency of occurrence indicated numerically within each grid cell.

To investigate the potential regulatory mechanisms of *GRAS* genes in *T. cuspidata*, cis-acting elements in the 2000 bp upstream promoter regions of 26 *TcGRAS* genes were systematically predicted and functionally categorized (Figure 4). A total of 89 cis-acting elements were identified across all TcGRAS family members, of which 43 were assigned putative functions. These elements were predominantly associated with abiotic stress, light-responsive regulation, phytohormone-responsive pathways, and plant growth and development. Elements related to abiotic and biotic stress comprised five distinct motifs: ARE, as-1, LTR, MBS, and WUN-motif. Phytohormone-responsive elements consisted of five categories: ABRE, CGTCA-motif, P-box, TCA-element, and TGACG-motif. Elements associated with plant growth and development included five elements: TATA-box, ERE, AAGAA-motif, CAT-box, and O2-site. Light-responsive elements were composed of five motifs: CAAT-box, Box 4, G-box, TCT-motif, and GT1-motif. Collectively, these findings suggest that *TcGRAS* genes may play crucial roles in the growth and development, hormone regulation, and stress adaptation of *T. cuspidata*.

### 3.6. Expression Patterns, GO Enrichment, and Protein–Protein Interaction Network Analysis of TcGRAS Genes

**Figure 5:**
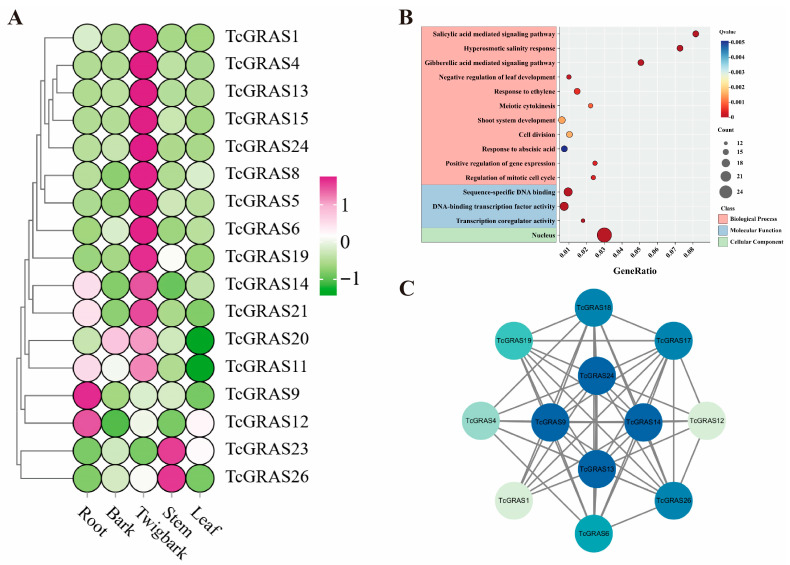
Expression patterns, GO enrichment, and protein–protein interaction network of *TcGRAS* genes. (**A**) Heatmap showing the expression of *TcGRAS* genes in roots, stems, leaves, twigs, and bark based on public transcriptome data. Color bars represent scaled relative expression levels, and the data in each heatmap correspond to raw FPKM values from the respective samples. (**B**) GO enrichment analysis of TcGRAS genes. (**C**) Protein–protein interaction network analysis of *TcGRAS* genes.

The expression patterns of *TcGRAS* genes were analyzed using transcriptomic data from five distinct tissues of *T. cuspidata*. The results revealed distinct tissue-specific expression patterns (Figure 5A). TcGRAS5, TcGRAS4, TcGRAS13, TcGRAS15, TcGRAS1, TcGRAS8, TcGRAS6, TcGRAS24, TcGRAS19, TcGRAS14, TcGRAS21, TcGRAS20, and TcGRAS11 were specifically upregulated in twig bark and were downregulated in root, stem, and leaf tissues. This coordinated expression pattern suggests a conserved functional role among these members. Likewise, TcGRAS12 and TcGRAS9 were exclusively upregulated in the root, whereas TcGRAS26 and TcGRAS23 were specifically upregulated in the stem tissue. These findings indicate that individual *TcGRAS* genes may play distinct biological roles across various tissues of *T. cuspidata*.

To further explore the functional properties of the *TcGRAS* gene family, Gene Ontology (GO) enrichment analysis was performed (Figure 5B), covering three categories: biological process, molecular function, and cellular component. In the biological process category, *TcGRAS* genes were predominantly enriched in pathways such as salicylic acid-mediated signaling, hyperosmotic salinity response, gibberellic acid-mediated signaling, negative regulation of leaf development, response to ethylene, positive regulation of gene expression, and regulation of the mitotic cell cycle. In the molecular function category, enrichment was observed in sequence-specific DNA binding, DNA-binding transcription factor activity, and transcription coregulator activity. In the cellular component category, genes were predominantly enriched in the nucleus.

To investigate potential co-regulatory interactions among *TcGRAS* genes, a gene co-expression network was constructed (Figure 5C). Twelve *TcGRAS* genes were identified as interacting within the network. Notably, *TcGRAS24*, *TcGRAS9*, *TcGRAS13*, and TcGRAS14 were positioned at central nodes, showing high degrees of connectivity and forming robust co-expression relationships with multiple other genes. These patterns suggest that they may serve as key regulators within the TcGRAS transcriptional network.

### 3.7. qRT-PCR Analysis of Tissue-Specific Expression Patterns of TcGRAS Genes

**Figure 6:**
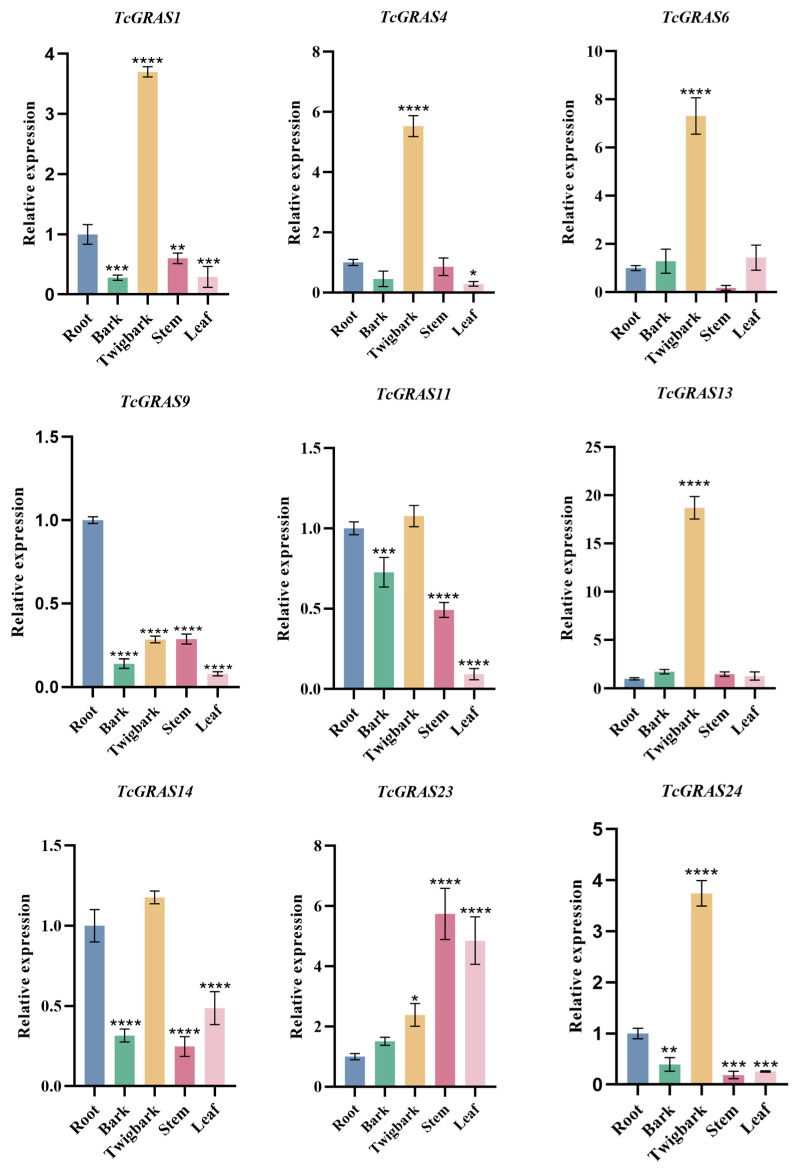
qRT-PCR analysis of *TcGRAS* gene expression across different tissues of *T. cuspidata*. Error bars represent the standard deviation of three biological replicates. Asterisks above the bars indicate statistically significant differences in expression levels relative to the Root. * *p* < 0.05, ** *p* < 0.01, *** *p* < 0.001, **** *p* < 0.0001.

To validate the accuracy of transcriptome-derived expression profiles, qRT-PCR was performed to quantify the expression profiles of nine representative *TcGRAS* genes across five tissues of *T. cuspidata*—root, bark, twig bark, stem, and leaf (Figure 6). The results demonstrated significant variation in gene expression among tissues, indicating pronounced tissue specificity. *TcGRAS1*, *TcGRAS4*, *TcGRAS6*, *TcGRAS13*, and *TcGRAS24* were highly expressed in twig bark, while expression levels in the root, stem, and other tissues were substantially lower. In contrast, *TcGRAS9*, *TcGRAS11*, and *TcGRAS14* exhibited higher expression levels in the root, whereas *TcGRAS23* was primarily expressed in the stem and leaf. These findings support the tissue-specific regulatory roles of *TcGRAS* genes and corroborate the transcriptomic data.

## 4. Discussion

As a relict gymnosperm, *T. cuspidata* has been recognized as the only natural source of paclitaxel. GRAS proteins, a plant-specific family of transcription factors, have been shown to play critical regulatory roles in diverse physiological processes, including growth and development, hormone signaling, and responses to abiotic stresses [22]. To date, members of the GRAS gene family have been identified and functionally characterized in various plant species, such as *Avena sativa* [23], *Raphanus sativus* [24], *Dendrobium catenatum* [6], *Zea mays* [25], *Eucalyptus grandis* [3], and *Medicago sativa* [4]. However, a comprehensive and systematic investigation of the GRAS gene family in *T. cuspidata* remains lacking.

### 4.1. Structural and Evolutionary Features of the GRAS Gene Family in T. cuspidata Exhibit Distinct Characteristics

Significant variability in the number of *GRAS* genes has been reported across plant species, with 34 in *A. thaliana* [26], 54 in tomato [27], 60 in rice [28], and up to 177 in wheat [29]. In contrast, only 26 *GRAS* genes were identified in *T. cuspidata*, a substantially lower count, potentially reflecting long-term functional integration and evolutionary conservation. This reduction is likely associated with the species’ complex genomic architecture and its gymnosperm lineage. Physicochemical analyses demonstrated substantial heterogeneity among TcGRAS proteins in terms of amino acid length, theoretical isoelectric point, instability index, and hydrophobicity, indicating pronounced structural and functional diversification. This finding is consistent with previous results in alfalfa [4], suggesting that structural polymorphisms within the GRAS family may contribute to the regulation of diverse physiological and developmental processes, including stress responses, tissue differentiation, and hormone signaling. Phylogenetic analysis further demonstrated that the 26 TcGRAS proteins clustered into six subfamilies, forming well-supported clades with homologs from *A. thaliana* (34 AtGRAS), *O. sativa* (60 OsGRAS), and *P. trichocarpa* (91 PtGRAS), indicating robust evolutionary conservation. Members within each subfamily shared similar gene structures and conserved motifs, underscoring both the functional stability and evolutionary inheritance of this gene family. Motif and domain analyses indicated that conserved functional regions were primarily located in the C-terminal domain, while significant divergence was observed in the N-terminal region. This structural flexibility may enable distinct TcGRAS members to adopt specialized regulatory functions and signaling responses, consistent with the structural variability previously described by Liu et al. [30]. Gene structure analysis showed that approximately 38.5% of *TcGRAS* genes lacked introns, and most of the remaining genes contained only one or two introns. This streamlined intron architecture, comparable to that observed in cucumber [31], may facilitate accelerated transcriptional responses under stress conditions [32].

### 4.2. Cis-Regulatory Element and Go Analysis Reveals the Multifunctionality of TcGRAS Genes

Cis-regulatory element analysis revealed that the promoter regions of *TcGRAS* genes were found to be enriched in regulatory motifs involved in light responsiveness, hormone signaling, growth and development, and abiotic stress responses. This enrichment suggests that *TcGRAS* genes are integrated into a complex transcriptional regulatory network and are likely to perform broad biological functions. In particular, the abundance of light-responsive elements in TcGRAS promoters was significantly greater than previously reported in other plant species [32], implying a more prominent role in light perception and photoadaptation. This likely reflects the ecological adaptation of *T. cuspidata* to understory, low-light environments in which the efficient regulation of photosynthesis is essential. Regarding developmental regulation, TATA-box elements were identified in all TcGRAS promoters, with notably high frequencies observed in TcGRAS1, TcGRAS6, and TcGRAS21 (64, 75, and 100 elements, respectively). This suggests a pivotal role in tissue-specific expression and developmental stage regulation and may contribute to the formation and secondary growth of structural tissues such as stems and twig bark. Moreover, promoter regions harbored multiple cis-elements responsive to abscisic acid (ABA), gibberellins (GA), methyl jasmonate (MeJA), and salicylic acid (SA), indicating that *TcGRAS* genes may participate in diverse hormone signaling pathways. For example, *GRAS* genes have been implicated in ABA-mediated drought resistance in poplar [33] and GA-regulated developmental processes in other species [34]. In terms of abiotic stress response, most TcGRAS promoters contained cis-elements related to hypoxia, drought, and salinity, suggesting roles in environmental adaptation and stress defense. Notably, certain genes such as TcGRAS23 were enriched with both hormone- and stress-responsive elements, exhibiting classic characteristics of signal crosstalk and potentially serving as integrators of external and internal signaling pathways.

GO enrichment analysis further indicated that several TcGRAS members were significantly associated with the salicylic acid-mediated signaling pathway. Previous studies have demonstrated that SA signaling promotes the expression of paclitaxel biosynthetic genes such as *TASY* [35], leading to the hypothesis that these *TcGRAS* genes may indirectly influence paclitaxel biosynthesis via SA signaling modulation. Although functional validation is still required, this finding offers new insights into the regulatory roles of GRAS transcription factors in hormone signaling and provides a potential entry point for elucidating upstream regulatory mechanisms of paclitaxel metabolism.

### 4.3. Functional Divergence Potential of TcGRAS Genes in T. cuspidate

Spatiotemporal expression analysis provides crucial insights into the potential biological functions of genes [21]. In this study, the spatiotemporal expression profiling of *TcGRAS* genes revealed distinct tissue-specific expression profiles across various organs of *T*. *cuspidata*, suggesting their multifunctionality and potential functional divergence during plant development and environmental adaptation. Notably, several TcGRAS members (TcGRAS5, TcGRAS4, TcGRAS13, and TcGRAS15) were highly expressed in the twig bark, the primary site of paclitaxel biosynthesis and accumulation [36]. This expression pattern implies that these genes may be involved in the regulation of vascular tissue development, thereby indirectly influencing paclitaxel synthesis or transport. Members of the HAM and SCR subfamilies of the GRAS gene family have been shown to be critically involved in meristem maintenance, cell division, and vascular differentiation [37]. Based on this evidence, the highly expressed *TcGRAS* genes are hypothesized to be involved in secondary growth, vascular formation, and xylem development, which may, in turn, affect paclitaxel biosynthesis via regulation of vascular development. Additionally, several *TcGRAS* genes displayed organ-specific expression profiles in other tissues. For example, TcGRAS9 and TcGRAS26 were predominantly expressed in roots, suggesting roles in regulating root development, nutrient uptake, or stress adaptation, thus potentially enhancing the adaptability of *T. cuspidata* to complex environmental conditions. This expression divergence supports the hypothesis that *TcGRAS* genes execute tissue-specific functions across different organs.

Further research is warranted to functionally characterize these highly expressed TcGRAS members through approaches such as CRISPR/Cas9-mediated gene editing or virus-induced gene silencing (VIGS), in order to elucidate their specific regulatory roles in the paclitaxel biosynthetic pathway.

## 5. Conclusions

In this study, 26 members of the GRAS transcription factor family were systematically identified and characterized in *T. cuspidata*, distributed across 10 chromosomes and classified into six distinct subfamilies. The *TcGRAS* genes exhibited strong evolutionary conservation, as evidenced by promoter regions enriched in cis-regulatory elements involved in light responsiveness, hormone signaling, and abiotic stress responses. Expression profiling and co-expression network analysis revealed that several genes—such as TcGRAS13, TcGRAS14, and TcGRAS24—were highly expressed in twig bark and occupied central positions within the co-expression network, indicating their putative involvement in paclitaxel biosynthesis via hormone-mediated regulatory pathways. Collectively, these findings provide a theoretical foundation and a valuable set of candidate genes for future functional studies of GRAS transcription factors in *T. cuspidata*, particularly in the context of secondary metabolism and paclitaxel biosynthesis.

## Figures and Tables

**Table 1:** Physicochemical properties of *TcGRAS* genes.

Sequence ID	Number of Amino Acids	Molecular Weight	Theoretical pI	Instability Index	Aliphatic Index	Grand Average of Hydropathicity
TcGRAS1	647	71,235.42	5.81	58.8	77.68	−0.378
TcGRAS2	177	19,725.61	5.15	34.31	87.01	0.081
TcGRAS3	261	28,649.41	4.73	40.51	95.98	−0.067
TcGRAS4	612	67,175.21	5.95	58.12	77.52	−0.468
TcGRAS5	596	63,669.06	5.62	49.64	68.15	−0.552
TcGRAS6	515	57,609.72	4.61	39.35	80.1	−0.33
TcGRAS7	561	63,439.37	4.56	46.86	77.02	−0.361
TcGRAS8	280	31,481.6	7.17	51.79	111.43	0.026
TcGRAS9	745	81,673.17	5.33	47.87	74.07	−0.306
TcGRAS10	156	17,703	5.96	51.48	86.22	−0.212
TcGRAS11	605	67,384.64	4.73	49.58	84.99	−0.375
TcGRAS12	557	62,434.98	5.47	52.91	81.83	−0.273
TcGRAS13	592	65,868.94	5.34	37.26	76.49	−0.507
TcGRAS14	385	43,992.3	5.53	42.25	81.06	−0.386
TcGRAS15	732	82,508.3	5.81	52.95	87.68	−0.243
TcGRAS16	477	54,182.12	5.78	37.72	89.37	−0.129
TcGRAS17	251	28,369.17	5.42	41.07	86.73	−0.223
TcGRAS18	230	26,586.59	6.67	53.56	90.26	−0.239
TcGRAS19	556	62,685.07	6.25	41.16	90.29	−0.036
TcGRAS20	499	56,327.17	4.97	45.77	87.33	−0.24
TcGRAS21	453	50,918.65	4.74	51.84	80.55	−0.189
TcGRAS22	152	17,072.55	5.93	48.32	95.53	−0.122
TcGRAS23	853	92,140.23	6	45.62	75.77	−0.446
TcGRAS24	629	69,349.67	5.54	48.57	73.66	−0.372
TcGRAS25	132	14,348.34	4.23	63.13	110.76	0.168
TcGRAS26	449	50,692.78	5.23	41.03	89.53	−0.125

## Data Availability

The original contributions presented in this study are included in the article/Supplementary Materials. Further inquiries can be directed to the corresponding author.

## Supplementary Materials

The following supporting information can be downloaded at: https://www.mdpi.com/article/10.3390/genes16111345/s1. Table S1: Primer sequences used for qRT-PCR.
