# Supplementary material for: Genome-Wide Identification and Expression Profiling of the GRAS Gene Family in Taxus cuspidate
Source: Genes (Basel). 2025 Nov 7;16(11):1345. doi: 10.3390/genes16111345 (PMC12652091; doi:10.3390/genes16111345)
Supplement: Supplementary file 1 [file genes-16-01345-s001.zip › Additional file 1.pdf]

Questionnaire

Dear participants,

Hello! We are conducting an academic study on how artificial intelligence technology can alleviate women's fertility anxiety, aiming to provide more scientific fertility support programs for women. We sincerely invite you to fill out this questionnaire based on your true feelings. Your answers will be strictly confidential and will only be used for academic statistical analysis. The questionnaire takes about 5-8 minutes to fill out, and there are no standard answers to all questions. Please feel free to answer. Thank you for your support and trust!

- Your age:

□ 18-25 years old

□ 26-35 years old

□ 36-50 years old

- Are you currently pregnant

□ Not giving birth (including preparing for pregnancy)

□ I have already given birth to one child

□ I have already given birth to two or more children

- Your professional status:

□ In enterprises/institutions

□ Liberal professions

□ Full-time housewife

□ Other______

- How often do you use AI products (such as smart assistants, health apps, etc)

□ Almost every day □ Several times a week □ Several times a month □ Rarely

□ Never

- Please choose the most appropriate option based on your actual feelings, from “strongly disagree" to "strongly agree". There is no right or wrong answer.

	strongly disagree	disagree	Neutral	Agree	Strongly agree
Worrying about the quality of the child					

The personalized parenting knowledge recommendations provided by artificial intelligence help alleviate my concerns about my child's health/education quality.					
By analyzing children's growth data (such as health indicators and learning abilities) through AI, I have more confidence in the quality of future parenting.					
The development risks of children predicted by AI, such as diseases and educational shortcomings, can help me develop response strategies in advance.					
Worrying about the time cost of childbearing					
AI tools, such as intelligent schedule management and prenatal examination process optimization, help me reduce time waste related to childbirth.					
AI's suggestions for dividing parenting tasks, such as household chores and partner collaboration, have reduced my time anxiety.					
The AI simulated fertility time planning (such as matching occupation and fertility nodes) has made my future arrangements clearer.					
worrying about the decrease in life quality					
The cost estimation of reproductive economy provided by AI (such as medical and educational expenses) has alleviated my financial anxiety.					
The personalized life balance plan recommended by AI, such as work flexibility and leisure activities, has improved the quality of life after childbirth.					
The optimization suggestions of AI for household resource allocation (such as housing and care support)					

make me feel more at ease.					
Worrying about intergroup emotions					
The AI-built fertility mutual aid community (such as experience sharing and emotional support) has alleviated my sense of loneliness.					
The family communication mode analyzed by AI (such as partner/elder interaction) helps me improve relationship conflicts.					
The emotional resonance training provided by AI, such as empathy dialogue simulation, has enhanced my ability to cope with social pressure.					
